# Prevalence of iron deficiency and red blood cell transfusions in surgical patients

**DOI:** 10.1111/vox.13194

**Published:** 2021-08-24

**Authors:** Rik Paulus Bernardus Tonino, Michael Wilson, Jaap Jan Zwaginga, Martin Roelof Schipperus

**Affiliations:** ^1^ Haematology Haga Teaching Hospital The Hague The Netherlands; ^2^ TRIP Haemovigilance and Biovigilance Office Leiden The Netherlands; ^3^ Haematology Leiden University Medical Centre Leiden The Netherlands; ^4^ Surgery Erasmus Medical Centre Rotterdam The Netherlands; ^5^ CCTR Sanquin Blood Supply Amsterdam The Netherlands; ^6^ Haematology University Medical Centre Groningen Groningen The Netherlands

**Keywords:** anaemia, iron deficiency, iron‐deficiency anaemia, iron parameters, patient blood management, perioperative management, red blood cell transfusion

## Abstract

**Background and Objectives:**

While iron deficiency (ID) is the most common cause of anaemia, little is known about the prevalence and type of ID in preoperative surgical patients. The aims of the present study were to investigate the prevalence and types of ID in a large cohort of surgical patients, and how these are related to perioperative blood use after correction for confounders such as haemoglobin level.

**Materials and Methods:**

Data were retrospectively extracted from electronic case records of all patients who underwent elective surgery between September 2016 and November 2017 (*n* = 2711). Iron parameters, haemoglobin and details of perioperative red cell transfusions were collected.

**Results:**

Of 2711 patients, 618 (22.8%) were iron deficient (= transferrin saturation [TSAT] < 16%) preoperatively, 173 (6.4% of the cohort) had an absolute iron deficiency (AID; TSAT < 16% and ferritin < 30 μg/L) and 445 (16.4%) had functional/mixed ID (TSAT < 16% and ferritin ≥ 30 μg/L). Corrected for Hb level, iron‐deficient patients received significantly more red cell units than patients without ID (*p* = 0.026). AID was not associated with a significantly higher incidence of transfusions (7.5% of patients transfused; *p* = 0.12 after correction for Hb) than patients without ID, whereas patients with functional/mixed deficiency did receive significantly more transfusions (6.1%; *p* = 0.021) as compared to patients without ID (1.7%).

**Conclusion:**

Preoperative ID, in particular the functional/mixed type, was associated with a higher risk of receiving perioperative red cell transfusions as compared to patients without ID. Adequately treating ID might, therefore, reduce the need for perioperative red cell transfusions.

## INTRODUCTION

### Background

Patient blood management (PBM) refers to the application of evidence‐based medical and surgical concepts to optimize the preoperative haemoglobin concentration and haemostatic potential and to minimize blood loss during surgery. PBM aims to improve patient outcome by improving low preoperative haemoglobin levels and reducing perioperative red blood cell transfusion (RBCT) support [1, 2, 3, 4, 5]. Correction of iron deficiency (ID)–associated anaemia is one of the most applied measures and has received much attention in recent years.

ID is commonly found in patients undergoing surgery and is associated with increased risk not only for receiving an RBCT but also of prolonged hospitalization and postoperative mortality and morbidity [6, 7, 8, 9, 10].

ID can either be an absolute ID (AID) due to blood loss or insufficient dietary intake, or functional, as a consequence of chronic inflammation leading to insufficient utilization of the iron stores and decreased uptake by the enterocytes [11]. In the case of functional ID, intravenous iron administration is often needed because of the poor enteric iron uptake.

The preoperative assessment of iron parameters is not standard practice in the Netherlands. In risk groups, in which iron parameters are assessed more often, the use of preoperative iron therapy has become a pragmatic standard of care, with orthopaedic, abdominal and cardiac surgery using the intravenous route of administration as the most effective and fast‐acting modality [1, 3, 12, 13, 14, 15].

### Objectives

Our objectives are to investigate the preoperative prevalence and type of ID and whether the different types of ID are associated with perioperative RBCTs. If such an association is found, the presence and type of ID will be relevant to define the target population in which to evaluate whether iron administration has an impact on perioperative RBTC and the clinical outcome. If the effect depends on the type of ID, this will allow the identification of patients who may benefit from iron supplementation in a cost‐effective way [7]. Therefore, we performed a retrospective study in a large cohort of surgical patients to investigate whether ID is associated with perioperative RBCTs, corrected for predefined confounders such as haemoglobin (Hb) and whether the transfusion requirement is additionally associated with the absolute or functional/mixed type of ID.

## METHODS

### Study design

This is a retrospective single‐centre study in the Haga Teaching Hospital, a large clinical referral centre in the Netherlands. The requirement for written informed consent was waived by the Leiden‐Den Haag‐Delft ethics committee. Permission was granted by the board of the Haga Teaching Hospital. The trial was performed in accordance with the Good Clinical Practice guidelines and the Declaration of Helsinki 2013.

### Study population

The study population was comprised of all adult patients who underwent any form of elective inpatient surgery between September 2016 and November 2017. Patients who underwent non‐elective or outpatient surgery or in whom iron parameters were not tested were excluded.

### Data collection

In September 2016, testing iron parameters (ferritin, transferrin, transferrin saturation [TSAT] and iron) preoperatively (<30 days before surgery) was introduced as the standard of care, but in November 2017, this practise was stopped due to cost reduction considerations. Iron parameters, Hb level, C‐reactive protein (CRP), administration of perioperative RBCTs (30 days before to 30 days after surgery), type of surgery, age and sex of patients were collated. Data were obtained from electronic medical records by the authors.

### Definition of anaemia, ID and classifications into subtypes of ID

In accordance with the WHO criteria, anaemia was defined as an Hb concentration of <13 g/dl for adult men and Hb < 12.0 g/dl for adult, non‐pregnant women [16]. With the collected data, we attempted to determine the origin of the ID (functional ID; AID and mixed). No universally accepted definitions of absolute, functional and mixed ID exist. Therefore, we chose to divide the cases into groups based on TSAT and ferritin. Reference values were taken from published literature, as indicated.

Patients were considered iron deficient when TSAT was <16% [17, 18]. In order to assess whether the type of ID was of influence on the need of RBCTs, we made a subdivision for all ID patients: a patient was considered to have AID when ferritin was <30 μg/L and not AID (FMID: functional/mixed ID) when ferritin was ≥30 μg/L [17, 19].

The prevalence of ID and the subtypes were calculated with the aforementioned criteria. We compared demographic data and perioperative RBCTs between patients with ID (AID and FMID) and non‐ID (TSAT ≥ 16%) to assess the association between ID and RBCTs.

### Statistical analysis

Categorical variables are summarized as frequencies and percentages, and compared with the chi‐square test; continuous variables are reported as means and standard deviations, and analysed with a one‐way analysis of variance.

We stratified our data by anaemia (binary) to evaluate additional value of iron parameters over Hb. Odds ratios are presented to show the association between preoperative ID and perioperative RBCTs. The Dunn–Bonferroni correction was used to compensate for multiple hypothesis testing. Ordinal regression modelling was carried out to explore the predictive value of iron parameters for RBCTs. In the ordinal regression model, the variables assessed included Hb level (in this case non‐binary), ferritin, TSAT, perioperative RBCTs, age, CRP, sex and type of surgery. All analyses were conducted using SPSS (version 25.0, SPSS Inc., Chicago, IL). A *p*‐value <0.05 (two‐sided) was considered statistically significant.

## RESULTS

### Inclusion and data collection

**FIGURE 1:**
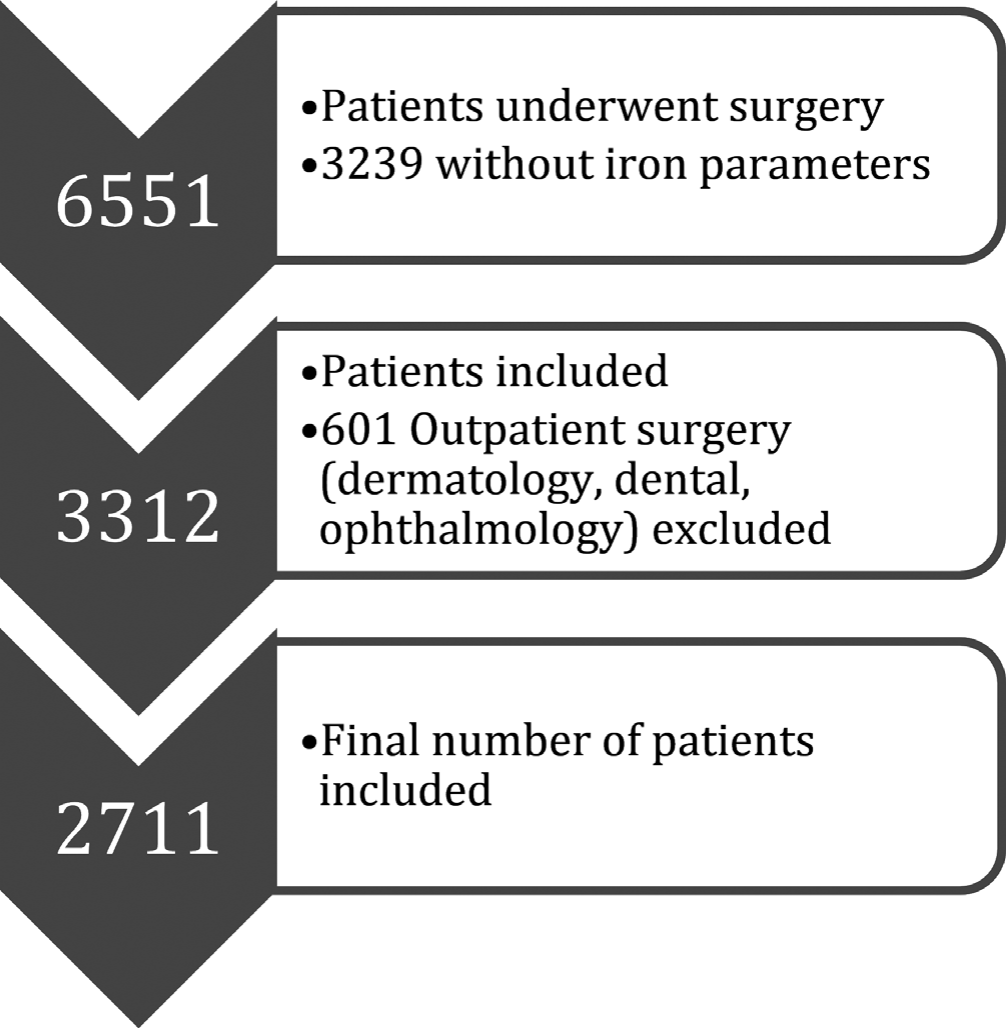
Inclusion of patients who underwent inpatient surgery. Patients were excluded if they did not have iron parameters tested or if they underwent an outpatient type of surgery

From September 2016 to November 2017, 6551 adult patients underwent surgery. Of these patients, 3239 had no iron parameters determined. The major part of the missing iron parameters can be attributed to non‐elective surgery, in which the testing of iron parameters was not standard care. Among the 3312 patients for whom iron parameters were available, 601 underwent outpatient surgery, like dermatologic or ophthalmologic and were excluded. Leaving 2711 who were included in our analyses (Figure 1). Iron parameters were tested <30 days before surgery (median = 21 days). Baseline characteristics of included patients are shown in Table 1.

**TABLE 1:** Patient demographics and perioperative variables

	AID	FMID	Non‐ID
Variables	(TSAT < 16% and ferritin < 30 μg/L) *n* = 173	(TSAT < 16% and ferritin > 30 μg/L) *n* = 445	(TSAT ≥ 16%) *n* = 2093
Demographics			
Age mean (±SD)	55 (±17)^a^ ^,^ ^b^	65 (±14)	64 (±13)
Female gender, *n* (%)	145 (84%)^a^ ^,^ ^b^	288 (65%)^a^	1173 (56%)
Preoperative blood analysis			
CRP, mean (±SD)	4.4 (±5.4)^b^	13,6 (±25.8)^a^	3,2 (±4.7)
Ferritin, mean (±SD)	17.9 (±7.0)^a^ ^,^ ^b^	160 (±163)^a^	197 (±171.3)
TSAT, mean (±SD)	9.2 (±3.6)^a^ ^,^ ^b^	12,7 (±2.6)^a^	25,3 (±7.4)
Anaemia, *n* (%)	95 (54.9%)^a^ ^,^ ^b^	105 (23.6%)^a^	163 (7.8%)
Mean Hb, g/dl (±SD)	11.9 (±1.6)^a^ ^,^ ^b^	13.2 (±1.6)^a^	14.0 (±1.3)
Perioperative RBCTs *n* (%)			
0 RBCT	160 (92.5%)	418 (93.9%)	2058 (98.3%)
1 RBCT	2 (1.2%)	4 (0.9%)	7 (0.3%)
2–3 RBCTs	7 (4.0%)	15 (3.4%)	22 (1.1%)
>3 RBCTs	4 (2.3%)	8 (1.8%)	6 (0.3%)

Abbreviations: AID, absolute iron deficiency (TSAT < 16% and ferritin < 30 μg/L); FMID, functional and mixed iron deficiency (TSAT < 16% and ferritin ≥ 30 μg/L; Hb, haemoglobin; non‐ID, non‐iron deficiency (TSAT ≥ 16%); RBCT, red blood cell transfusion; TSAT, transferrin saturation.

^a^ *p* < 0.05 compared to non‐ID;

^b^ *p* < 0.05 compared to FMID.

### Prevalence of ID and anaemia

**FIGURE 2:**
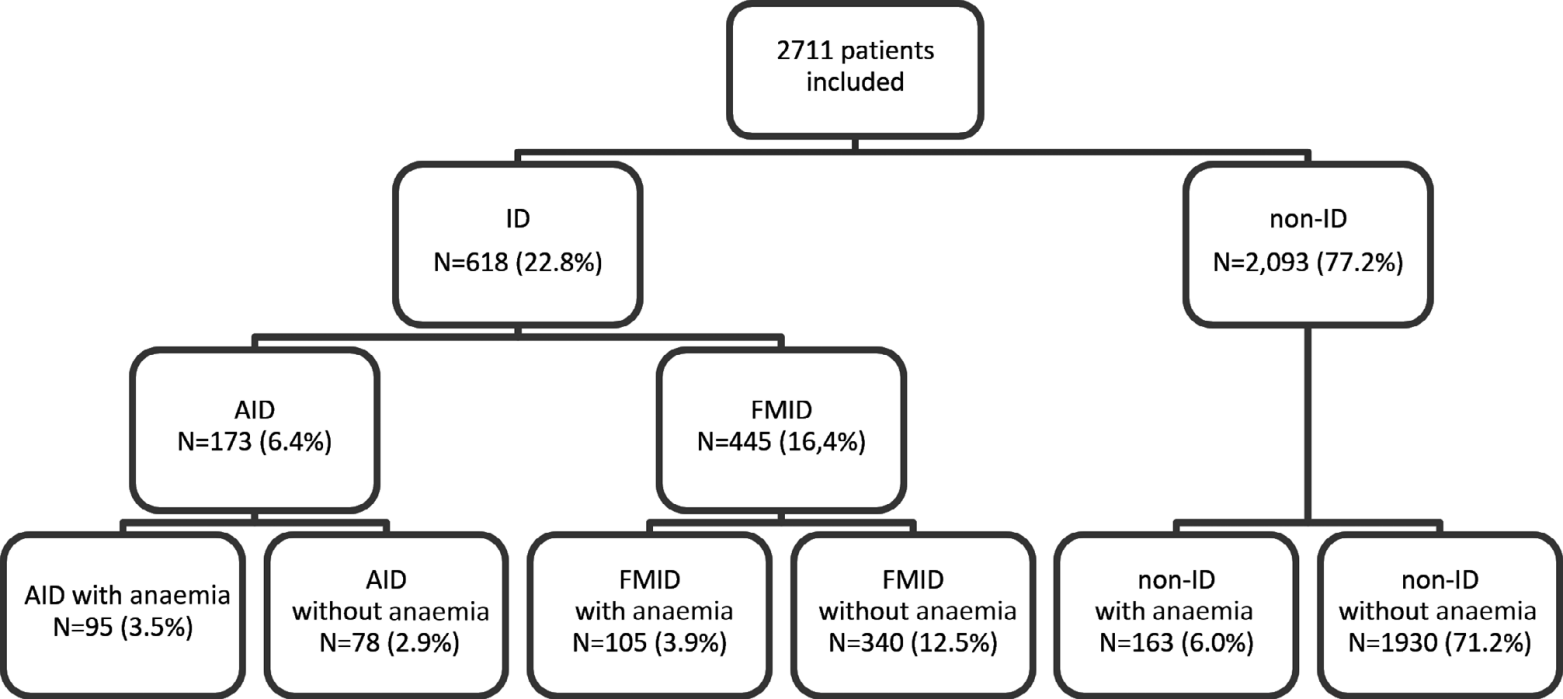
Prevalence of iron deficiency in the preoperative population. ID, iron deficiency (TSAT < 16%); AID, absolute iron deficiency (TSAT < 16% and ferritin < 30 μg/L); FMID, functional and mixed iron deficiency (TSAT < 16% and ferritin ≥ 30 μg/L; non‐ID, non‐iron deficiency (TSAT ≥ 16%); TSAT, transferrin saturation

Of patients 2711, 618 (22.8%) had ID preoperatively, 173 (6.4%) had AID and 445 (16.4%) had FMID. Of the 618 patients with ID, 32.4% were anaemic (54.9% of patients with AID and 23.6% of FMID). In the group without ID, 7.8% were anaemic (Figure 2). Conversely, among the 363 (13.4%) anaemic patients, 95 (26.2%) had AID and 105 (28.9%) FMID (Table 1). For anaemic patients, the mean Hb was 11.0 ± 1.1 g/dl in the ID group (AID: 10.8 ± 1.3 g/dl; FMID: 11.3 ± 0.8 g/dl) and 11.7 ± 0.6 g/dl in the non‐ID group.

**FIGURE 3:**
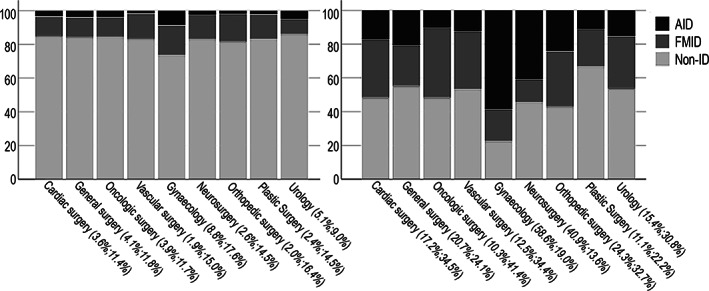
Iron deficiency per field of surgery, subdivided in non‐anaemic patients (a) and anaemic patients (b). (% of patients with AID and FMID per surgical group are given). AID, absolute iron deficiency (TSAT < 16% and ferritin < 30 μg/L); FMID, functional and mixed iron deficiency (TSAT < 16% and ferritin ≥ 30 μg/L; non‐ID, non‐iron deficiency (TSAT ≥ 16%); TSAT, transferrin saturation

The prevalence of preoperative ID for the different surgical specialties is shown in Figure 3. As can be expected, in each surgical field, the prevalence of ID is significantly higher in anaemic patients than in non‐anaemic patients. The prevalence of AID was highest at 20.3% in gynaecological surgery patients (51/251).

### ID and red cell transfusions

Overall, including both anaemic and non‐anaemic patients, we found that patients with ID receive more RBCTs than non‐ID patients (6.5% and 1.7%, resp.; *p* < 0.001). This is the case for both AID: 7.5% (13/173) and FMID: 6.1% (27/445; *p* < 0.001 for both types, compared to non‐ID) (Table 2).

**TABLE 2:** Number of RCTs in patients with and without iron deficiency, anaemic and non‐anaemic patients separately

	AID	FMID	Non‐ID	
No anaemia	(TSAT < 16% and ferritin < 30 μg/L) *n* = 78	(TSAT < 16% and ferritin > 30 μg/L) *n* = 340	(TSAT ≥ 16%) *n* = 1930	Total *n* = 2348
No RBCT	76 (97.4%)	331 (97.4%)	1904 (98.6%)	2311 (98.4%)
1 RBCT	0	2 (0.6%)	5 (0.3%)	7 (0.3%)
2‐3RBCTs	0	5 (1.4%)	17 (0.9%)	22 (0.9%)
>3 RBCTs	2 (2.6%)^a^	2 (0.6%)	4 (0.2%)	8 (0.3%)
RBCT odds	2/76 (2.63%)	9/331 (2.72%)	26/1904 (1.37%)	37/2311 (1.60%)

Anaemia	*n* = 95	*n* = 105	*n* = 163	*n* = 363
No RBCT	84 (88.4%)	87 (82.9%)^a^	154 (94.5%)	325 (89.5%)
1 RBCT	2 (2.1%)	3 (2.9%)	2 (1.2%)	6 (1.7%)
2‐3RBCTs	7 (7.4%)	9 (8.6%)	5 (3.1%)	22 (6.1%)
>3 RBCTs	2 (2.1%)	6 (5.7%)	2 (1.8%)	10 (2.7%)
RBCT odds	11/84 (13.10%)	18/87 (20.69%)^a^	9/154 (5.84%)	38/325 (11.69%)

Abbreviations: AID, absolute iron deficiency (TSAT < 16% and ferritin < 30 μg/L); FMID, functional and mixed iron deficiency (TSAT < 16% and ferritin ≥ 30 μg/L; non‐ID, non‐iron deficiency (TSAT ≥ 16%); RBCT, red blood cell transfusion; TSAT, transferrin saturation.

^a^ *p* < 0.05 compared to non‐ID; no significant differences were found comparing AID to FMID in this table.

Evaluating the surgical specialties separately, we found that more patients in cardiac surgery (*p* = 0.003) and orthopaedics (*p* < 0.001) receive RBCTs when they have ID as compared to patients of the same specialties who do not have ID. In the other surgical specialties, a comparable trend was found, but no significant difference in RBCTs between ID and non‐ID patients.

### ID, anaemia and patients receiving RBCTs

We stratified for the presence and absence of anaemia to see whether the association between ID and RBCTs remained. Of all patients with neither ID nor anaemia, 1.35% (26/1930) received RBCTs. In patients with ID but without anaemia, 2.6% (11/418) received RBCTs (2.6% and 2.7% for AID and FMID, respectively). Of the patients with ID and anaemia, 14.5% (29/200) received RBCs (11.6% and 17.1% for AID and FMID, respectively) while among patients with anaemia but no ID, 5.5% (9/163) were transfused perioperatively (Table 2).

The odds ratio for receiving RBCTs in iron‐deficient patients versus non‐iron‐deficient patients is 1.99 (Confidence interval (CI): 0.97–4.05) for non‐anaemic patients and 2.90 (CI: 1.33–6.32) for anaemic patients.

### ID and number of RBCTs, corrected for Hb level

RBCTs were initially analysed as a binary variable (no RBC units vs. ≥1 units). Subsequently, we grouped patients according to the number of RBC units (0, 1, 2–3 or > 3 units as an ordinal variable) to evaluate whether the number of RBC units per transfused patient differed between the various groups. Because a severely anaemic patient (e.g., Hb = 6.0 g/dl) is more likely to receive one or more RBC units than a mildly anaemic patient (e.g., Hb = 12.0 g/dl), we also evaluated the influence of Hb level as a continuous variable (instead of the binary presence or absence of anaemia) among other possible confounders using ordinal regression.

After correction for Hb level, sex and age, ID was still correlated with a significantly larger number of RBC units transfused compared to non‐ID (*p* = 0.026). In the subgroup analysis, AID was not independently associated with a larger number of RBCTs than non‐ID (*p* = 0.12), whereas patients with FMID did receive more RBCTs than non‐ID (*p* = 0.021).

## DISCUSSION

In this study, we show that 22.8% of patients have ID preoperatively. Having ID resulted in a four‐fold increase in RBCT in our cohort. While anaemia is more often present in ID patients, stratification for anaemia shows that RBCTs are only transfused significantly more in ID patients if they are also anaemic. In the non‐anaemic group, there is a non‐significant trend of increased RBCT in ID patients.

Interestingly, the ordinal regression model indicated that patients with FMID received significantly more RBCTs compared to non‐ID, whereas patients with AID did not. This could be a first indication that we may need to specifically target FMID patients in order to cost‐effectively improve PBM. However, these findings may also be caused by underpowering, as the AID group (*n* = 173) is smaller than the FMID group (*n* = 445). Moreover, gynaecology patients have AID more often. Inclusion of gynaecology patients—who are more likely to have AID and anaemia, especially when pre‐menopausal [20]—might potentially be a confounding factor. These patients may often be young and healthy enough to tolerate anaemia well, and therefore, do not need transfusions, which would lead to a lower transfusion rate in the AID group. Exclusion of the gynaecology patients from our analyses, however, yielded the same results. Given the difference between FMID and AID, distinguishing between these types of ID may prove to be useful in the context of PBM [21, 22].

A recent observational study found that intravenous (IV) iron preoperatively reduced the risk of perioperative RBCTs [23]. In line with our results, Hubert et al. also found associations in elective cardiac surgery patients between ID and anaemia and between ID and the number of RBCTs (*p* = 0.03) perioperatively [24]. However, they did not correct the latter analysis for the Hb level, nor was the type of ID taken into consideration. Our findings, in contrast, clearly show a relation between preoperative ID and perioperative RBCTs after correction for the Hb level.

A smaller study of 100 patients undergoing cardiac surgery showed results consistent with ours: patients with ID but without anaemia received more RBCTs than patients with neither ID nor anaemia [25]. Conflicting with the latter and our findings, Fotland et al. did not find such a correlation in a group of 175 orthopaedic patients [26].

A small randomized controlled trial in cardiac surgery did not find a reduction in RBC transfusions after oral and IV iron [12]. However, the iron supplementation started when patients were admitted just before surgery, which may very well be too late for effective iron therapy. Another small trial in the UK with anaemic patients undergoing colorectal cancer surgery found that IV iron had no effect on perioperative blood use compared to oral iron supplementation [14], while a small Australian trial found that IV iron for iron‐deficient anaemic patients did improve perioperative blood use [3]. The larger PREVENTT trial randomized major open elective abdominal surgery patients to IV iron or placebo. Despite not selecting patients for ID, the authors showed the efficacy of IV iron for improving the Hb level by the time of surgery and at 8 weeks and 6 months following the intervention. Nevertheless, they concluded that there was no reduction in perioperative RBC transfusions, but there was a reduced risk in readmissions to the hospital for complications in the group that received IV iron [27]. It has been argued, however, that without a standardized approach to transfusion it should not be concluded that improving preoperative red cell mass does not reduce the need for blood transfusion [28].

In contrast to the above‐mentioned studies, the present study spanned the whole scope of surgery in a real‐life setting, which strengthens the external validity of our results. In general, we found a clear association between ID and the number of RBCTs after correction for predefined confounders, the most obvious being Hb level, but also age and sex. Other possible confounders (e.g., CRP, type of surgery) were also assessed to maximize the chance of finding a true effect.

Our study is limited, first, by its retrospective nature. The patients received standard care, which makes selection bias unlikely. However, we lack data about perioperative iron supplementation in our cohort, and iron parameters and Hb were not measured immediately before surgery, but any time in the 30 days before. This could lead to an underestimation of the effect because RBCTs may have been avoided in the group most at risk for receiving RBCTs, for example, if they received IV iron supplementation. We also lack information on perioperative blood loss. Having this data, as well as Hb and iron parameters measured precisely before surgery would have made our dataset and results more complete. Second, our data contain some outliers—for example, one patient in cardiac surgery received 43 RBCTs. The use of RBCTs as a continuous variable would have resulted in unbalanced data with a high impact of such outliers. Therefore, the number of RBC units transfused was used as an ordinal variable (0, 1, 2–3 or > 3 RBCTs) instead of a continuous variable. Third, even though we included 2711 patients, RBCT support was limited to only 77 patients (39 who were anaemic and 38 non‐anaemic). This obviously leads to a lack of statistical power. Last, the real‐life dataset did not comprise enough iron parameter testing results to properly distinguish between absolute, mixed and functional ID, forcing us to merge the mixed and function ID into the FMID group.

To find a conclusive answer to the question of whether IV iron preoperatively truly lowers the need for perioperative RBCTs, large prospective randomized controlled trials in various surgical fields are needed. Based on our data, such a trial would need at least 127 anaemic patients per group to acquire enough transfusion events to conclude on the effects of preoperative iron supplementation. For non‐anaemic patients, the number would need to be many times larger. An additional and more important question to resolve is whether iron supplementation with an increased Hb in consequence also leads to the hoped‐for better short‐ and long‐term outcomes. Although a lower Hb level and a higher amount of blood use are both correlated with inferior outcomes, we should keep in mind that this is likely to be confounded by the severity of the underlying pathology in these patients. Therefore, it remains unclear whether Hb correction by iron supplementation improves outcome. Until this is established, we recommend IV iron over oral iron when aiming to correct ID preoperatively. Particularly, because we found a stronger association of ID with the incidence of transfusions in the FMID patients, in whom the enteric uptake is impaired [11]. In addition, we recommend starting treatment at least 1 week before surgery so that the therapy has sufficient time to exert a beneficial effect.

In conclusion, our data show that preoperative ID, corrected for Hb level, is clearly associated with the number of RBCTs given to patients. As the association of FMID with the need for RBCTs is stronger than that of AID, our data support distinguishing between the types of ID. Further research should be undertaken to assess the potential and cost‐effectiveness of adequate ID correction preoperatively for reducing RBCTs. Treating ID even in patients without anaemia might also contribute to reduced need for perioperative RBCTs.

## CONFLICT OF INTEREST

All authors attest that they have no conflict of interest to declare.
